# Efficacy and confounding factors of CT attenuation value differences in distinguishing acute and old vertebral compression fractures: a retrospective study

**DOI:** 10.1186/s12891-023-06484-w

**Published:** 2023-05-10

**Authors:** Limin Liang, Ya Wang, Yaya Zhao, Chunyuan Luo, Jianghua Zhu, Xin Zhang, Zhaotao Zhang, Yinquan Ye, Weiwei Deng, Yun Peng, Lianggeng Gong

**Affiliations:** 1 Department of Radiology, The Second Affiliated Hospital of Nanchang University, Nanchang, 330006 Jiangxi China; 2 Clinical and Technical Support, Philips Healthcare, Shanghai, 200072 China

**Keywords:** Attenuation value (Hounsfield unit), Vertebral compression fractures, CT

## Abstract

**Purpose:**

To evaluate the influence of various factors on CT attenuation values (HUs) of acute and old fracture vertebra, and to determine the efficacy of HU differences (△HUs) in the differentiation of the two type of fractures.

**Materials and Methods:**

A total of 113 acute and 71 old fracture vertebrae confirmed by MRI were included. Four HUs measured at the mid-sagittal, upper 1/3 axial, mid-axial, and lower 1/3 axial planes of each vertebra were obtained. The △HUs between fracture vertebra and its control counterpart was calculated. Receiver operating characteristic (ROC) curve analysis was used and the areas under the ROC curve (AUC) were calculated to evaluate the efficacy of HUs and △HUs. To evaluate the effect of height reduction, region, age and gender on HUs and △HUs, one-way analysis of variance, Pearson correlation analysis and t-test were used.

**Results:**

The HUs and △HUs at the upper 1/3 axial plane achieved the highest AUCs of 0.801 and 0.839, respectively. The HUs decreased gradually from Thoracic to Lumbar in control group of acute fracture. While no significant differences were found in the HUs among the 3 localizations in both fracture groups (all *P* > 0.05). The HUs were negatively correlated with age in all groups. The HUs of male were significantly higher than female patients in all groups (all *P* < 0.05). While △HU was not significantly different between males and females (all *P* > 0.05).

**Conclusion:**

The vertebral HUs at the upper 1/3 axial plane are more likely to identify acute fractures. △HUs were beneficial in eliminating interfering factors.

## Introduction

As the incidences of osteoporosis and traumatic accidents have increased, vertebral compression fractures (VCFs) have become a common disease in the daily diagnostic work of radiologists, imposing a substantial burden on society [1, 2]. A fast and convenient method is necessary to distinguish incidental acute vertebral fracture from old fractures, as this would aid in treatment decision making-and the reduction of later complications [3]. Magnetic resonance imaging (MRI) is currently the gold standard for identifying acute and old fractures and can help diagnose specific types of fractures, such as occult VCFs. However, MRI examinations in some primary hospitals are not available under the hierarchical medical mode [4]. Furthermore, MRI is not the preferred method for fracture patients without a history of serious trauma [5, 6].

Given its high-density resolution, computed tomography (CT) is a routine diagnostic modality for exploring the etiology of chest/abdominal pain [7]. The differential diagnosis of acute and old fractures by conventional CT scans can not only simplify the diagnostic process and reduce the economic burden on the patient and medical insurance but also compensate for the lack of MRI devices in primary medical institutions. Some studies have used a semiquantitative conventional CT parameter, the attenuation values (HUs), to evaluate acute occult fractures, since the accompanying marrow edema may cause an increase in bone marrow density [8, 9]. Recent studies have further predicted fracture age by the value of trabecular attenuation [10]. However, these studies only measured HUs at a single plane/position and did not assess the influence of various factors on the diagnosis.

We hypothesized that factors such as the method for measuring HU, individual factors (including sex and age) and vertebral condition (including localization and degree of vertebral height loss) might influence the evaluation process. Therefore, the purpose of this study was to evaluate the above potential confounding factors on HUs in VCF patients, and further validate performance of the HU differences (△HU), which is obtained from the difference between the fractured and the adjacent control vertebra, in distinguishing acute and old fractures.

## Materials and methods

### Study population

The current study was a retrospective observational cohort study. The ethics committee approved the study and the waived the need for informed consent [2019] No.(077). All methods were carried out in accordance with relevant guidelines. This study included patients with acute/old VCFs who underwent chest and/or abdominal CT examination between August 2019 and February 2022 in our hospital. All of the VCFs were confirmed by MRI within 72 h. The exclusion criteria included (1) diffuse metabolic bone disease, malignant tumors that may cause bone metastasis, spinal tumors, and spinal infections; (2) Schmorl’s nodule in the target vertebrae; (3) no adjacent normal vertebrae to the fractured vertebrae in the same region; (4) severe compression that prevented measurement of the HUs of the fractured vertebrae at different axial levels; and (5) artifacts on the raw CT images.

### Imaging acquisition

All CT scans were performed with two CT scanners, a Philips Brilliance 256 iCT and a Brilliance 16-Slice scanner. The collimations of the scanners were 256 × 0.625 mm and 16 × 0.75 mm, respectively. the kilovoltage peak were 120 KVp, and the tube current time product were 90 to 170 mAs. The image thickness of the original axial images was 1.0 mm, with a matrix of 512 × 512 mm. For each patient, a single image in the mid-sagittal plane of the spine was saved in DICOM format.

MRI scans were performed with a 1.5 T MRI device (Signa Hdx; GE Healthcare) using a dedicated spine surface coil. The imaging protocol comprised a sagittal T1-weighted fast spin echo sequence and a short-tau inversion-recovery sequence.

### Vertebrae selection

The diagnosis and classification of VCFs were determined by a radiologist with 18 years of experience according to MRI, CT and medical history [11]. Acute fractures were determined according to the presence of bone marrow edema with or without loss of vertebral height. Old fractures were identified based on a history of trauma, loss of vertebral height, and absence of marrow edema. Once a fractured vertebra was included, an adjacent normal vertebra from the same region was selected as a control.

The spine was divided into 3 regions: thoracic spine (T, Th1-Th10), thoracic-lumbar junction (TLJ, Th11-L1) and lumbar spine (L, L2-L5) [12].

Then, the fractured vertebrae were divided into 4 grades according to the Genant semiquantitative grading scheme [13]: grade 0 (normal or uncertain, height loss < 20.0%), grade 1 (mild, height loss 20.0–24.9%); grade 2 (moderate, height loss 25%-40%), or grade 3 (severe, height loss ≥ 40%).

### Measurements of vertebral CT attenuation

**Fig. 1:**
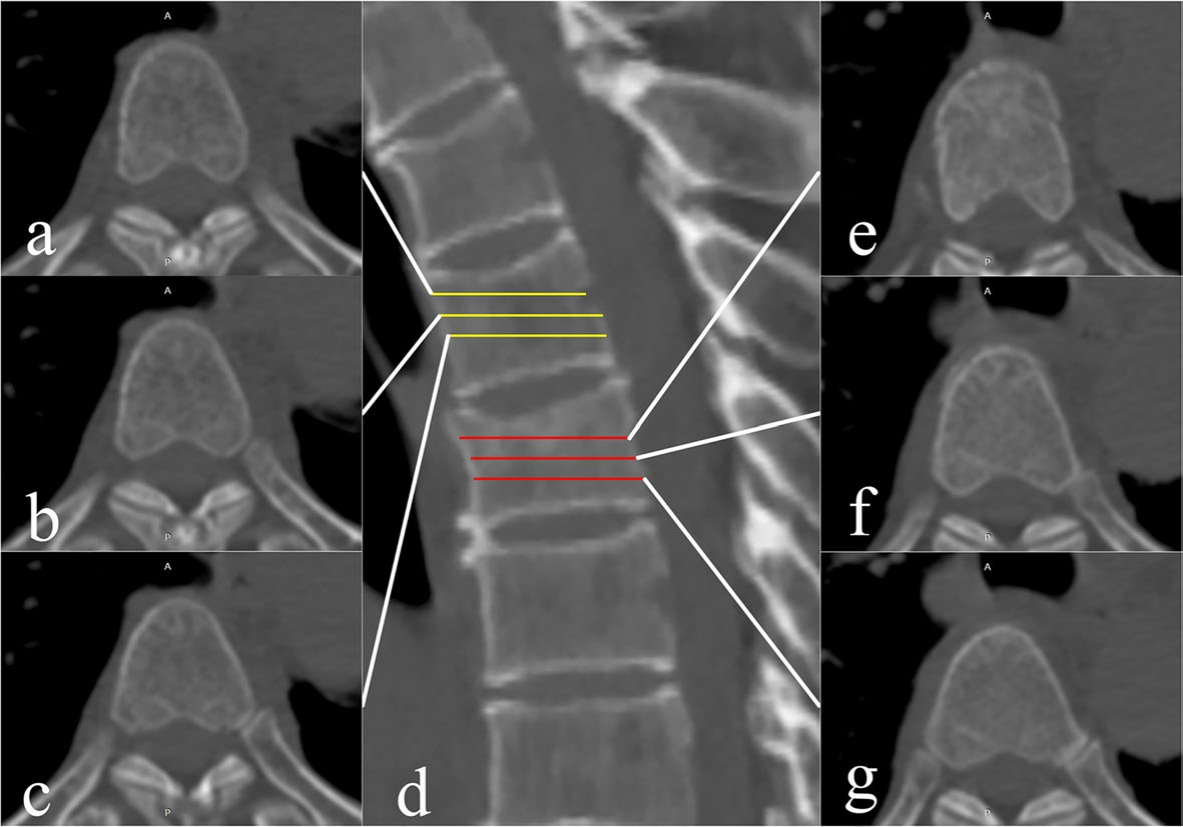
Diagram of CT attenuation value measurements for vertebra. **d** shows the mid-sagittal image; T5 was an acute fracture vertebra confirmed by MRI; and T4 was selected as the control vertebra. Quadrilateral ROIs were used in this plane for CT value measurement. **a**, **b**, and **c** show the upper 1/3 axial, mid-axial, and lower 1/3 plane of T4, for which oval ROIs were used. **e**, **f**, **g** show the corresponding measurement planes of T5. The differences and ratios between the HUs between T5 and T4 were calculated

Two radiologists (with 18 and 5 years of clinical experience) who were blinded to the MRI findings obtained four HUs by placing regions of interest (ROIs) in four positions for each vertebral body: the mid-sagittal plane, the upper 1/3 axial plane, the mid-axial plane, and the lower 1/3 axial plane (Fig. 1). When delineating the ROIs, the readers were blinded to the MRI, the clinical information and radiological reports and included as much area as possible while keeping a minimal distance of 4 mm from the cortex. The mean values of the two radiologists were used for subsequent analysis. The CT attenuation value differences (△HUs) and ratios between the fracture vertebrae and the corresponding adjacent control vertebrae were calculated.

### Statistical analysis

Statistical analysis was performed in SPSS 19.0 and GraphPad Prism 7. Whether the data conform to normal distribution was evaluated by the Kolmogorov‐Smirnov test. The Mann–Whitney U test was used to compare the ages of patients with acute fractures and those with old fractures. The chi-square test was used for comparisons of composition ratios (sex, Genant grade, and localization of vertebrae) between groups. For interobserver agreement analysis between the two radiologists, the intraclass correlation coefficient (ICC) was calculated.

To evaluate the differences in HUs under different measurement methods, one-way analysis of variance (ANOVA) was used to compare the HUs of the acute fracture, old fracture and control groups in the mid-sagittal, the upper 1/3 axial, the mid-axial, and the lower 1/3 axial planes. The independent-samples t test was used to compare the differences in the HUs between two groups. We further used receiver operating characteristic (ROC) curve analysis to evaluate the effectiveness of the HUs, △HUs and ratios in differentiating among the acute fracture, old fracture and control groups. The area under the ROC curve (AUC) was obtained.

To evaluate the effect of the height reduction on the HUs, one-way ANOVA was used to compare the differences in HUs among different Genant grades in the acute fracture, old fracture and their corresponding control groups. ROC analyses were performed to evaluate the effectiveness of HUs and △HUs in distinguishing acute and old fractures with different grades. Pairwise comparisons were performed with the least significant difference (LSD) method. To evaluate the effect of vertebrae localization on the HUs, one-way ANOVA was used to compare the vertebral HUs of the T, TLJ and L regions in the four groups, further pairwise comparisons were performed.

The influence of personal factors was only evaluated using the HUs of the upper 1/3 axial plane, given their high diagnostic efficiency and stability. Pearson’s correlation analysis was used to evaluate the correlation between HUs and age in four groups. To evaluate the effect of sex on vertebral HUs, the independent samples t test was used to compare the HUs of males and females in each group.

Finally, to evaluate whether the HUs and △HUs from different CT machine images were different, the independent samples t tests were conducted.

## Results

### Patient characteristics

A total of 184 vertebrae (including 113 acute fracture vertebrae and 71 old fracture vertebrae) and 184 adjacent normal vertebrae from 147 patients were included in the study. Among them, 109 patients were enrolled with 1 pair of vertebrae, 25 with 2 pairs of vertebrae, 7 with 3 pairs of vertebrae, and 1 with 4 pairs of vertebrae. There were no significant differences in age, sex, or region between the acute fracture group and the old fracture group, but there were significant differences in Genant grades (Table 1). The ICC of the HUs measured by the two radiologists at the mid-sagittal, upper 1/3 axial, mid-axial and lower 1/3 axial planes of the vertebrae were 0.955, 0.969, 0.973, and 0.984, respectively.

**Table 1:** Clinical characteristics of the acute fracture and old fracture groups

	Acute fracture group (*n* = 113)	Old fracture group (*n* = 71)	chi-square /F value	*P* value
Age	64 (53.5,73)	69 (57,76)	-	0.53
sex			1.173	0.279
Male	57 (50.44%)	30 (42.25%)		
Female	56 (49.56%)	41 (57.75%)		
Region			0.018	0.991
T	28 (24.78%)	17 (23.94%)		
TLJ	57 (50.44%)	37 (52.12%)		
L	28 (24.78%)	17 (23.94%)		
Genant grade			18.463	< 0.001^*^
0	42 (37.17%)	9 (12.68%)		
1	25 (22.12%)	11 (15.49%)		
2	27 (23.89%)	29 (40.84%)		
3	19 (16.82%)	22 (30.99%)		

### Influence of measurement method on the HU and △HU

The HUs measured in the 4 planes are shown in Table 2. One-way ANOVA indicated that the HUs of the 4 measurement methods were significantly different in the acute fracture group (F = 4.319, *P* = 0.005). However, there was no significant difference among the HUs in the other 3 groups (F = 1.188, *P* = 0.314; F = 1.015, *P* = 0.387; F = 0.828, *P* = 0.479). Pairwise analysis indicated that the HUs at the mid-sagittal plane and the upper 1/3 axial plane were significantly higher than those at the lower 1/3 axial plane (*P* = 0.009, *P* = 0.001).

**Table 2:** CT attenuation values (HUs) measured using different methods in four groups

	Mid-sagittal	Upper 1/3 axial	Mid-axial	Lower 1/3 axial	F values	*P* values
Acute fracture group	168.25 ± 70.46	174.88 ± 70.11	157.79 ± 65.52	144.68 ± 63.31	4.319	0.005*
Control group A	104.69 ± 49.70	95.15 ± 48.61	104.18 ± 47.91	96.38 ± 50.23	1.188	0.314
Old fracture group	101.73 ± 58.50	101.95 ± 61.16	95.61 ± 54.20	87.75 ± 49.16	1.015	0.387
Control group B	82.81 ± 5.23	77.74 ± 40.80	83.55 ± 42.20	73.87 ± 41.46	0.828	0.479

^*^Control group A, control group of acute fracture group; Control group B, control group of old fracture group

The four kinds of HUs of the acute fracture group were statistically higher than those of the control group and old fracture group (all *P* < 0.001). The HUs of the old fractures at the mid-sagittal plane and the upper 1/3 axial plane were significantly higher than those of the corresponding control group (*P* = 0.031, *P* = 0.006). There were no statistical differences between the HUs of the old fracture group and the control group at the mid-axial plane and lower 1/3 axial plane (*P* = 0.141, *P* = 0.071).

**Fig. 2:**
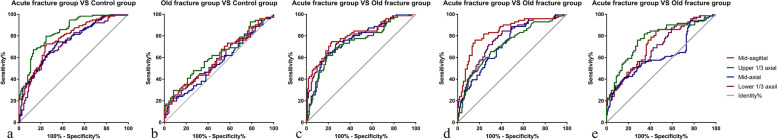
ROC curves of the CT attenuation values (HUs), value difference (△HUs) and ratio at the 4 positions in differentiating fracture and control groups. 2**a** ~ 2**c** shows the ROC curves of the HUs measured at the 4 planes in discriminating the acute fracture from control group, the old fracture from control group, and the acute fracture from the old fracture group. 2**d** ~ 2**e** shows the ROC curve for the △HUs and ratio in differentiating the acute and old fracture groups

ROC curve analysis showed that the AUCs of the HUs measured at the mid-sagittal, the upper 1/3 axial, the mid-axial, and the lower 1/3 axial planes in differentiating acute fracture vertebrae from their control vertebrae were 0.778, 0.840, 0.749, 0.728, respectively (Fig. 2a). The AUCs of the HUs in discriminating old fracture vertebrae from their control vertebrae were 0.592, 0.618, 0.550, 0.514, respectively (Fig. 2b). The AUCs of the HUs in differentiating acute from old fractures were 0.779, 0.801, 0.737, 0.761, respectively (Fig. 2c). The AUCs of the △HUs in distinguishing acute and old fractures were 0.773, 0.839, 0.757, 0.727, respectively (Fig. 2d). The AUCs of the ratio were 0.721, 0.773, 0.618, 0.699, respectively, which was lower than △HU (Fig. 2e). The parameters at the upper 1/3 axial plane showed the best performance.

### Influence of vertebral factors on HUs and △HUs

**Fig. 3:**
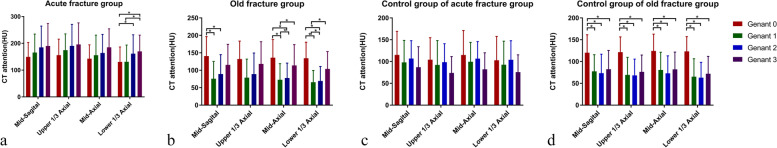
CT attenuation values of vertebrae with different Genant grades measured in different positions in the acute fracture group (3**a**), old fracture group (3**b**), and the corresponding control group of acute fracture (3**c**) and control group of old fracture (3**d**). * Indicates pairwise differences

The HUs of fractured vertebrae of different Genant grades and corresponding control vertebrae are shown in Fig. 3. For the control group of acute fracture, one-way ANOVA showed no significant differences among different grades (F = 1.616, *P* = 0.190; F = 1.828, *P* = 0.146; F = 2.236, *P* = 0.099; F = 1.596, *P* = 0.195). While the HUs of Genant grade 0 vertebrae were significantly higher than those grades 1, 2, and 3 vertebrae for the control group of old fracture (all *P* < 0.05). For the acute fracture group, the HUs on the mid-sagittal plane, upper 1/3 axial plane and mid-axial plane showed no differences among the different Genant grades (F = 2.193, *P* = 0.093; F = 2.104, *P* = 0.104; F = 2.012, *P* = 0.116). However, significant differences were found in the HUs of the lower 1/3 axial plane (F = 2.837, *P* = 0.041). Further pairwise comparison indicated that the HUs of grade 3 vertebrae were higher than those of grades 0 (*P* = 0.024) and grade 1 vertebrae (*P* = 0.043). The HUs of grade 2 vertebrae were higher than those of grade 0 vertebrae (*P* = 0.045). For the old fracture group, there was no difference in the HUs on the upper 1/3 axial plane among different grades (F = 2.326, *P* = 0.083), but there shown difference at other positions (*P* < 0.05).

The AUCs for HUs differentiation between the two fracture groups were 0.598, 0.887, 0.904, 0.782, for Genant grades 0, 1, 2, 3, respectively. While, the diagnostic performance of △HUs were all increased compared with HUs, with AUCs 0.817, 0.953, 0.923, 0.883, respectively.

**Fig. 4:**
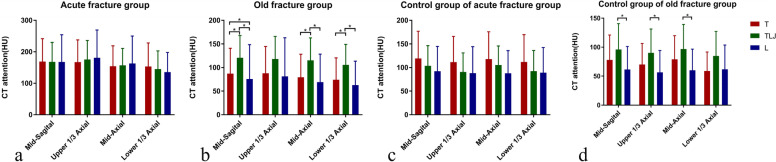
CT attenuation values of vertebrae of different regions at the four planes in the acute fracture group (4**a**), old fracture group (4**b**), the corresponding control group of acute fracture (4**c**) and control group of old fracture (4**d**). * Indicates pairwise differences

Figure 4 shows the HUs for vertebrae of different regions measured at the 4 planes in the different groups. For the control group of acute fracture, HUs showed a gradual downward trend from T to L regions. One-way ANOVA indicated that there was no difference in the HUs for the 3 different localizations in the acute fracture group (all *P* > 0.05). For the control group of old fracture, the HUs on the mid-sagittal, upper 1/3 axial, and mid-axial planes were significantly different in different regions (F = 4.210, *P* = 0.019; F = 4.610, *P* = 0.013; F = 5.059, *P* = 0.066). Pairwise comparison showed that the HUs of the T and TLJ regions were higher than those of the L region. For the old fracture group, the HUs at the upper 1/3 axial plane showed no significant difference among the 3 localizations (F = 2.864, *P* = 0.064), while significant differences were found in the HUs at the other 3 planes among localizations.

### Influence of personal factors on HU and △HU

Pearson correlation analysis showed that the HUs in the acute fracture group, old fracture group and their control groups were negatively correlated with age (*r* = -0.590, *P* < 0.001, *r* = -0.555, *P* < 0.001, *r* = -0.742, *P* < 0.001, *r* = -0.540, *P* < 0.001).

The HUs and the △HUs of males and females are shown in Table 3. The HUs of male patients were higher than those of female patients in all groups (*P* < 0.001, *P* = 0.004, *P* < 0.001, *P* < 0.001). There was no significant difference between the △HUs of males and females in the two VCF groups.

**Table 3:** CT attenuation values (HUs) and the HU differences (△HUs) of female and male cases

	HUs of acute fracture group	HUs of control group A	△HUs of acute fracture group	HUs of old fracture group	HUs of control group B	△HUs of old fracture group
Female	156.14 ± 64.50	78.03 ± 41.37	78.101 ± 51.36	77.87 ± 48.58	61.86 ± 36.16	16.01 ± 34.59
Male	193.29 ± 71.05	111.97 ± 49.64	81.32 ± 55.70	134.86 ± 61.91	99.44 ± 37.03	35.42 ± 46.08
*P* value	0.004*	< 0.001*	0.751	< 0.001*	< 0.001*	0.058

Control group A, control group of acute fracture; Control group B, control group of old fracture

### Influence of CT machine on HUs and △HUs

There were some numerical differences in HUs measured from different CT machine images in each group, among which there were significant differences in the control group for old fractures (*P* = 0.028). While there were no significant differences between the △HU of two machines in other groups (Table 4).

**Table 4:** CT attenuation values (HUs) and the HU differences (△HUs) from two CT machines

	HUs of acute fracture group	HUs of control group A	△HUs of acute fracture group	HUs of old fracture group	HUs of control group B	△HUs of old fracture group
CT1	190.18 ± 61.41	107.06 ± 52.57	83.11 ± 49.15	88.30 ± 49.70	64.19 ± 28.33	24.11 ± 38.83
CT2	170.97 ± 71.95	92.11 ± 47.38	78.86 ± 54.63	108.09 ± 65.20	83.82 ± 44.24	24.26 ± 41.91
*P* value	0.243	0.189	0.739	0.210	0.028*	0.989

CT 1 Philips Brilliance 16-Slice, CT 2 Philips Brilliance 256 iCT

Control group A, control group of acute fracture; Control group B, control group of old fracture

## Discussion

To the best of our knowledge, our study was the first to evaluate the efficacy of HUs measured at multiple positions and the impact of individual and vertebral characteristics on VCF evaluation, through which we draw the following conclusions. Among the 4 measurement positions, the HU of the upper 1/3 axial plane had the highest AUC in differentiating acute from old fractures. The localization and vertebral height loss, individual age and sex, CT machine showed varying degrees of influence on the HUs. The △HU between the target vertebra and the adjacent vertebra in the same region may play a role in avoiding the influence of these factors.

Our study found that the HUs of both the acute and old fracture vertebrae were higher than those of normal vertebrae, and the HUs of acute fractures were significantly higher than those of the other groups. The bone structure disruption, overlap and cancellous edema caused by acute VCFs resulted in increased density as well as HUs, consistent with previous studies [8]. The increase in the HUs of old fractures was less than that in the acute stage, which may be related to the complete absorption of marrow edema.

The HUs measured at the upper 1/3 axial plane had the best performance in distinguishing acute and old fractures. A reasonable explanation may be that the patients included in this research were mainly elderly individuals, whose VCFs were mostly caused by weight bearing or slight stress. The main stress point is always on the upper edge of the vertebral body [14]; hence, the incidence of edema and bone overlap involving the upper 1/3 portion is the highest. This is supported by the fact that the increase in HUs in the mid-axial and lower 1/3 axial planes was less than that in the upper 1/3 plane. Moreover, the results suggested that we should focus on the changes in HUs to exclude occult fractures in consideration of the high frequency of this phenomenon in Genant grade 0 VCFs. Yan et al.found that the quantitative analysis with the virtual noncalcium technique based on dual-energy CT performed well in identifying acute and old VCFs [3]. Our highest AUC is close to that of their study (0.839 vs. 0.851), indicating the need to improve the measurement method.

Most previous studies have rarely considered the influence of different vertebral body-related factors in identifying the type of VCFs [10]. The HUs of the control vertebrae of Genant grade 0 old fracture were higher than those of grades 1, 2, and 3 vertebrae, which may be related to the higher proportion of males (grades 0, 1, 2, 3, 7/9, 5/11, 9/29, 9/22) and relatively young age (55.89, 65.18, 72.76, 65.45 years old). Nevertheless, significant differences were found only in the HUs of the lower 1/3 axial plane among the different Genant grades in the acute VCF group. The HUs of vertebrae with greater height reduction were higher than those with mild collapse. This may be related to the greater of height reduction, the greater degree of edema and bone overlap involving the lower part of the vertebrae, which is reflected by the higher degree of HUs increase. There were no differences in the HUs at the upper 1/3 axial plane among the different grades in the old fracture group, the reason could be the bone overlap in the upper part of vertebrae exist in most fractures.

In the control group of acute fracture, the HUs decreased from the T to the L regions. We speculated that decreased functional mobility with impaired vertebral strength in the elderly population might alter mechanical loading and lead to bone loss, predominantly affecting heavier weight-bearing vertebrae accompanied by lower localization [15]. While the HUs of TLJ were higher than T, the higher proportion of males (T, TLJ, L, 6/17, 19/37, 5/17) might be the reason. Nevertheless, there was no significant differences among the different regions in the acute fracture group due to the common factors that increased the HUs. No significant difference was found in the HUs at the upper 1/3 axial plane among old VCFs in different regions. The reason for this phenomenon may be related to the high probability of fracture involving the upper 1/3 portion mentioned above. After the absorption of marrow edema, the pathological changes, including bone remodeling are common. Overall, a significant difference was found in the HUs of normal vertebrae in different regions, but the HUs measured at the upper 1/3 plane after VCFs (for both acute and old fractures) were relatively stable.

With increasing age, the HUs of normal vertebrae gradually decreased, this could be relevant for age-associated bone loss [16]. The vertebral HUs of males were higher than those of females in both fracture groups and the control groups. The reason may be that bone loss in women rapidly increases after menopause due to lower levels of estrogen [17]. We also noticed that vertebral HUs had large individual differences. In some patients, the HUs of the control vertebral body were close to 0 HU, the HUs of their acute fracture vertebrae were even lower than HUs of other patuents’ control vertebrae. Moreover, the HUs of the control group corresponding to old VCFs were lower than those of the acute VCFs, which might be a result of the combination of relatively older age (65.89 vs. 67.18) and a higher proportion of males (57/113 vs. 30/41) in the old fracture group. Finally, we found that there were some differences in HUs measured from different CT machine images. Due to the different scanning parameters and signal conversion mode of different machines, the attenuation of photons through objects is different [18]. It is for the purpose of eliminating these potential influencing factors that we calculated the △HUs and ratio between the HUs of the fractured and the normal vertebra in the same region of the same person. The AUCs of ratios is lower than △HUs, so the other data of ratios was not exhibited. These results suggest that the use of the △HUs between the target vertebral body and the control can effectively remove various potential influencing factors, which is beneficial to discriminate acute and old fractures.

This study had some limitations. First, this study focused on the potential influencing factors in the process of distinguishing acute and old fractures by the quantitative index, HUs, and did not analyze other conventional imaging features. We expect to further combine multiple signs to achieve better results on the basis of this experiment. Second, the presence of bone marrow edema on MRI was used as the gold standard for the classification of fracture type. However, a small minority of VCFs may generate little or no marrow edema in acute injury, potentially leading to false negative results in MRI. Third, considering the extensibility of the research results, the axial HUs were measured on the original axial CT images. While the axial images of the body may not be the axial images of each vertebral body due to the existence of physiological spinal curvature or pathological spinal curvature changes. As a result, the HUs were measured at approximate slices rather than the expected standard plane, which is more in line with the actual situation of imaging reading. Furthermore, although there was no significant difference in age, sex or region between the two fracture groups, the effect of multiple factors superimposed inevitably interfered with the results. This is why the control vertebral HUs of Genant grade 0 VCFs were higher than those of grades 1, 2, and 3 VCFs. Further large-scale and multicenter evaluations are needed.

In conclusion, HUs can reflect the increased bone density caused by marrow edema in acute VCFs and bone overlap in old VCFs to a certain extent. Considering that potential interference factors, including individual or vertebral conditions, may affect the HU, the △HU obtained by selecting a suitable control vertebra for further evaluation could help eliminate the influence of confounding factors. This finding may be helpful for primary medical institutions that lack of MR machines and for patients who cannot undergo MR examination.

## Data Availability

The datasets generated and/or analysed during the current study are not publicly available due [Data security], but are available from the corresponding author on reasonable request.
